# Genetic profiling of multidrug-resistant *Acinetobacter baumannii* from a tertiary care center in Malaysia

**DOI:** 10.1128/spectrum.00872-24

**Published:** 2024-12-20

**Authors:** Aisyah Syakirah Shahari, Navindra Kumari Palanisamy, Fadzilah Mohd Nor

**Affiliations:** 1 Institute for Medical Molecular Biotechnology (IMMB), Faculty of Medicine, Universiti Teknologi MARA, Sg. Buloh Campus, Jalan Hospital, Sg. Buloh, Selangor, Malaysia; 2 Department of Medical Microbiology and Parasitology, Faculty of Medicine, Universiti Teknologi MARA, Sg. Buloh Campus, Jalan Hospital, Sg. Buloh, Selangor, Malaysia; 3 Integrative Pharmacogenomics Institute (iPROMISE), Universiti Teknologi MARA, Puncak Alam Campus, Puncak Alam, Selangor, Malaysia; College of New Jersey, Ewing, New Jersey, USA

**Keywords:** multidrug resistant, *Acinetobacter baumannii*, antibiotic resistance genes, sequence types, evolution

## Abstract

Genetic characterization of multidrug-resistant (MDR) *Acinetobacter baumannii* remains scarce in Malaysia. This study aimed to characterize antibiotic resistance, genomic location, and genetic relatedness among the *A. baumannii* isolates obtained from a tertiary hospital in Malaysia. A total of 128 MDR *A. baumannii* isolates were collected from patients admitted to various wards (intensive care unit [ICU], neonatal intensive care unit, coronary care unit, high dependency ward [HDW], and general wards). The isolates were identified by Vitek 2 and PCR amplification of the 16S rRNA gene followed by sequencing. The isolates were tested against imipenem, ceftazidime, amikacin, gentamicin, ampicillin, and ciprofloxacin using disk diffusion, Epsilometer test, and broth microdilution. The antibiotic resistance genes, *bla*_OXA-23_, *bla*_OXA-24_, *bla*_ADC_, *bla*_VIM,_ and *bla*_IMP_, were detected in chromosomal and plasmid DNA using PCR. Insertion sequence IS*Aba*1/*bla*_OXA-23_ gene was detected on chromosomal DNA only. Isolates with different antibiotic susceptibility patterns and PCR profiles were subjected to multi-locus sequence typing. MDR *A. baumannii* was predominantly found in HDW (39.84%), general wards (29.69%), and ICU (28.13%). All isolates conferred resistance to carbapenem and more than 90% resistance to the remaining antibiotics. The antibiotic resistance genes *bla*_OXA-23_, *bla*_VIM,_ and *bla*_ADC_ were detected in both chromosomal and plasmid DNA. The IS*Aba*1/*bla*_OXA-23_ gene was detected in 99.22% of the isolates. Four sequence types (STs) were distinguished: ST2 (76.67%), ST164 (10%), ST642 (10%), and ST643 (3.33%). ST164 and ST642 were unique and represent a significant finding in Malaysia’s surveillance data. These STs are associated with acquired *bla*_OXA-23_, indicating an evolutionary adaptation of *A. baumannii* within the hospital setting.

**IMPORTANCE:**

*Acinetobacter baumannii* is a ubiquitous Gram-negative coccobacillus bacterium that is primarily associated with nosocomial infections that can colonize biotic and abiotic surfaces to enhance cell-to-cell adhesion, ensuring the establishment of infections. To date, the spread of multidrug-resistant *A. baumannii* (MDRAB) has become rampant and a great concern in the hospital setting, as the available antibiotics are insufficient to treat infections. The antibiotic resistance island resides in a mobile element and rapidly evolved. The antibiotic susceptibility data with its resistance mechanisms would contribute to and facilitate the management and infection control caused by MDRAB.

## INTRODUCTION

There is a decreasing trend of susceptibility among *Acinetobacter baumannii* isolates to commonly used antibiotics, owing to escalating cases of multidrug-resistant (MDR) *A. baumannii*. Turkey and Romania were reported to have the highest incidence rate of imipenem resistance (95%) followed by Italy (93%) and Greece (89%), while the prevalence rate of imipenem-resistant *A. baumannii* in Asian countries is as follows: Vietnam (92%), India (85%), Iran (82%), and Saudi Arabia (72%) (1). The SENTRY Antimicrobial Surveillance Program (2020–2021) reported carbapenem resistance rates of *A. baumannii* as 26.0% in the United States and 61.8% in European countries including Turkey and Israel (2). The imipenem resistance rate in African countries such as Morrocco, Algeria, and Nigeria was more than 70% (3). For Southeast Asia, carbapenem-resistant *A. baumannii* (CRAB) has been reported to range from 1.76% to 64.91% (4), and several reports have shown that the prevalence of CRAB in Singapore, Thailand, and Vietnam ranges from 70.5% to 91.0%, 46.7% to 80.0%, and more than 90%, respectively (5). In Malaysia, according to the National Antibiotic Resistance Surveillance 2022 report, the resistance rate of MDR *A. baumannii* against several antibiotics increased drastically from 1987 to 2022, which includes imipenem (4.8%–62.4%) and amikacin (14.3%–48.9%) (6, 7).

There are three major resistance mechanisms in *A. baumannii,* which include the production of inactivating enzymes (β-lactamases), overexpression of the efflux pump, and modification of the target site. Class A β-lactamases or extended-spectrum β-lactamases (ESBL) confer resistance to penicillin and cephalosporin, while metallo-β-lactamases (MBL) harbor resistance against carbapenem, cephalosporin, and penicillin. The MBL-encoding genes, such as *bla*_IMP_, *bla*_VIM,_*bla*_NDM,_*bla*_KPC,_ and *bla*_SIM,_ are commonly located on plasmid DNA. The *bla*_VIM_ and *bla*_IMP_ genes are the most predominant and have many variants. It was documented that 27% of the *A. baumannii* isolates carried the *bla*_VIM-1_ gene in Saudi Arabia (8), while 9.9% of the *bla*_IMP_ gene was detected in *A. baumannii* isolates in Malaysia (9). Several studies on the emergence of chromosomally encoded *bla*_ADC_ genes (Class C β-lactamases) have been reported (10, 11). The Class C β-lactamases commonly encode for cephalosporin and penicillin resistance. In *A. baumannii*, insertion sequence (IS) is found throughout the bacterial genome and acts as a strong promoter upstream of the gene leading to the upregulation of the gene expression. The insertion of IS*Aba*1 upstream of the *bla*_ADC_ gene may increase the production of β-lactamases, causing resistance to cephalosporins. In Malaysia, the presence of the *bla*_ADC_ gene with IS*Aba*1 was reported in 93.7% of *A. baumannii* isolates (9). The Class D β-lactamases confer resistance to carbapenem, oxacillin, methicillin, and cloxacillin. This is attributed to the presence of genes, such as *bla*_OXA-23_, *bla*_OXA-48,_*bla*_OXA-24,_*bla*_OXA-51_, *bla*_OXA-143,_ and *bla*_OXA-58_, which spread either via plasmid or chromosomal DNA. Several ISs, including IS*Aba*1, IS*Aba*3, and IS*Aba*2, are responsible for enhancing the regulation of OXA-type β-lactamases genes in *A. baumannii* (12). Reports from Turkey and China showed a high prevalence of the *bla*_OXA-23_ gene with 96.5% and 100%, respectively (10, 13). In 2017, Malaysia reported the *bla*_OXA-23_ gene to be predominant as it was detected in approximately 95% of the carbapenem-resistant *A. baumannii* isolates (14). This trend is consistent with global data particularly in China and India (10, 15).

In addition, the overproduction of efflux pumps in *A. baumannii* often contributes to the resistance of quinolone, chloramphenicol, carbapenem, tigecycline, and tetracycline (16). It decreases the concentration of β-lactam antibiotics in the periplasmic space leading to the rejection of antibiotics out of the target. Four main families of efflux pumps have been identified in *A. baumannii,* including small multidrug resistance, major facilitator superfamily, resistance-nodulation cell division (RND), and multidrug and toxic compound extrusion, with RND being the most prevalent system in MDR *A. baumannii* (17). The AdeABC is the first identified RND efflux pump system and has a three-component structure comprising *AdeC, AdeB,* and *AdeA* (18). Overexpression of the AdeABC efflux pump harboring carbapenem-hydrolyzing oxacillinases leads to high-level carbapenem resistance. Furthermore, mutation in antibiotic target sites can hinder the binding of drugs and cause fluoroquinolone resistance in *A. baumannii* through modification in *parC* and *gyrA* genes (19).

As the emergence of MDR *A. baumannii* strains in the hospital setting is intensifying at an alarming rate, this study determined the genetic relatedness among the strains found in various wards incorporated with an elucidation of the genetic evolution among the circulating strains in the hospital setting. With this effort, an appropriate platform that assists infection control and management of MDR *A. baumannii* is established. Furthermore, it may serve as a significant molecular epidemiological tool for national and sentinel surveillance.

## MATERIALS AND METHODS

### Sample collection

#### Bacterial collection and hospital setting

A total of 128 MDR *A. baumannii* isolates were obtained from the Microbiology Laboratory, Department of Pathology, Hospital Sungai Buloh, Selangor, Malaysia between August 2017 and April 2018. The isolates were collected from patients admitted to the intensive care unit (ICU), coronary care unit (CCU), neonatal intensive care unit (NICU), high dependency ward (HDW), and general wards. The type of specimens includes tracheal aspirate (TRAS), bronchoalveolar lavage (BAL), blood, cerebrospinal fluid (CSF), urine, sputum, and others. All specimens were cultured on MacConkey agar and incubated at 37°C for 18–24 hours. The isolates were then sub-cultured and stored in Brain Heart Infusion broth supplemented with 10% glycerol and kept at −80°C until further use.

Three MDR *A. baumannii* isolates obtained from the Department of Microbiology, Universiti Kebangsaan Malaysia Medical Centre (UKMMC) in Kuala Lumpur, Malaysia, with reference numbers, MB10070709, MB11019933, and MB10065144, were used as positive controls in this study. These positive control isolates harbored multiple antibiotic resistance genes comprising *bla*_OXA-23_, *bla*_OXA-24_, *bla*_ADC_, *bla*_VIM_, *bla*_IMP^,^_ and IS*Aba*1.

### Bacterial identification and validation

The identification of isolates was performed using the VITEK 2 Gram-negative identification card system. The primer targeting the 16S rRNA region specific to *Acinetobacter* spp. was used to verify the identity of bacterial isolates of *Acinetobacter* to species level. Before performing PCR amplification, the DNA was extracted using boiling method and purified. The PCR amplification of the 16S rRNA gene of *Acinetobacter* spp. was performed using a commercial master mix as per the manufacturer’s protocol (SMOBIO Technology, Inc., Taiwan). The PCR products were separated using gel electrophoresis, and analysis was performed via GelDocXr + 446 System (Bio-Rad Laboratories, USA) and Image Lab Analysis Software (Thermo Scientific, USA). The PCR products were sent for DNA sequencing services (1st BASE Laboratories, Malaysia).

### Antimicrobial susceptibility testing

Three methods were used to determine the antimicrobial susceptibility testing (AST), including Kirby-Bauer disk diffusion, Epsilometer test (E-test), and broth microdilution. Bacterial suspension equivalent to 0.5 McFarland standard was lawned onto a Muller-Hinton agar (MHA) and six antibiotic disks; ampicillin (10 µg), ceftazidime (30 µg), gentamicin (10 µg), amikacin (30 µg), ciprofloxacin (5 µg), and imipenem (10 µg) were placed onto the MHA for disk diffusion and E-test, respectively. The agar plates were incubated at 37°C for 18–24 hours. The diameter of clear zones around the disk and inhibition of growth intersecting the E-test strip were measured and interpreted according to the 2023 Clinical and Laboratory Standards Institute (CLSI) guideline (20). In this study, imipenem was chosen as the representative carbapenem due to its stability to most β-lactamases (including ESBL), widespread use, and effectiveness as a first-line treatment for *A. baumannii* infections. The resistance patterns for imipenem are often indicative of broader carbapenem resistance in *A. baumannii* isolates (20–22).

For the broth microdilution method, cation-adjusted Mueller Hinton Broth (Sigma-Aldrich, USA) was used according to CLSI guidelines (20). Serial dilutions of five antibiotics including ceftazidime, gentamicin, amikacin, ciprofloxacin, and imipenem (256–0.06 µg/mL) were prepared. *A. baumannii* isolates were adjusted to 0.5 McFarland standard and added to 96-well microdilution plates. The plates were incubated at 35°C for 20–24 hours. *Pseudomonas aeruginosa* ATCC 27853 strain was used as a quality control. The minimum inhibitory concentration (MIC) was defined as the lowest concentration of antibiotic with no visible growth and interpreted according to CLSI guidelines (20).

### Antibiotic resistance gene detection

#### PCR amplification of antibiotic resistance genes

Conventional PCR was performed to detect antibiotic resistance genes, *bla*_OXA-23_, *bla*_OXA-24_, *bla*_ADC_, *bla*_VIM_, and *bla*_IMP_, in both chromosomal and plasmid DNA, while insertion sequence IS*Aba*1/*bla*_OXA-23_ gene detection was carried out on the chromosomal DNA only. The list and details of primer sequences are attached in the supplemental material (Table S1). The chromosomal DNA was extracted using the boiling method, while plasmid DNA was extracted using Monarch Plasmid Miniprep Kit (New England Biolabs, USA) according to the manufacturer’s instructions. The purified chromosomal and plasmid DNA were quantified using SpectraMax QuickDrop Micro-Volume Spectrophotometer (Molecular Devices, LLC) at wavelengths *A*_260_ and *A*_280_. The extracted DNA was stored at −20°C until further use. The PCR amplification, gel electrophoresis, and PCR sequencing were performed accordingly using similar methods mentioned in the bacterial identification and validation section.

### Multi-locus sequence typing

Of 128 MDR *A. baumannii* isolates, only 30 isolates were subjected to multi-locus sequence typing (MLST) owing to different antibiotic susceptibility profiles phenotypically as well as genotypically. The MLST analysis of the *A. baumannii* isolates was performed based on the sequence analysis of the internal fragments of seven housekeeping genes, including *cpn60, fusA, gltA, pyrG, recA, rplB,* and *rpoB*. All seven housekeeping genes were amplified and sequenced. The DNA sequencing results were aligned according to Pasteur’s MLST schemes from the PubMLST website (23). Comparative analysis of molecular sequences of the isolates was aligned using Molecular Evolutionary Genetics Analysis X Software (24). The estimation of isolate relatedness distance was generated using the unweighted pair-group method with the arithmetic averages method based on the MLST allelic profile (25). All sequence types (STs) obtained from MLST in this study were compared to established STs worldwide from the GenBank database (CP143348.1, CP042556.1, CP007577, CP050403.1, and CP029610.1).

## RESULTS

### Distribution and frequency of *A. baumannii* isolates

A total of 128 *A*. *baumannii* isolates were collected in this study. The isolates were mainly from HDW (39.84%), followed by general wards (29.69%) and ICU (28.13%). The *A. baumannii* isolates were predominantly isolated from respiratory secretion, such as TRAS (37.50%), followed by blood (19.53%), BAL (13.28%), and CSF (9.37%). The distribution and frequency of *A. baumannii* are shown in Table 1. All details on the collection date, source of specimens, and location of all *A. baumannii* isolates were included in the supplemental material (Table S2).

**TABLE 1:** Distribution of the clinical specimens and locations^*a*^

	2017	2018
Ward (*N* = 128)	*n* = 66	*n* = 62
HDW, 51 (39.84%)	26 (39.40%)	25 (40.32%)
General wards, 38 (29.69%)	17 (25.76%)	21 (33.87%)
ICU, 37 (28.13%)	21 (31.82%)	15 (24.20%)
NICU, 2 (1.56%)	1 (1.51%)	1 (1.61%)
CCU, 1 (0.78%)	1 (1.51%)	0
Specimen type (*N* = 128)	*n* = 66	*n* = 62
TRAS, 48 (37.50%)	24 (36.35%)	24 (38.71%)
Blood, 25 (19.53%)	13 (19.70%)	12 (19.35%)
BAL, 17 (13.28%)	9 (13.64%)	8 (12.90%)
CSF, 12 (9.37%)	11 (16.66%)	1 (1.61%)
Urine, 7 (5.47%)	3 (4.55%)	4 (6.45%)
Tissue, 7 (5.47%)	1 (1.52%)	6 (9.68%)
Pus, 5 (3.91%)	3 (4.55%)	2 (3.23%)
Others, 7 (5.47%)	2 (3.03%)	5 (8.07%)

^*a*^ HDW: High dependancy ward, ICU: intensive care unit, NICU: neonatal intensive care unit, CCU: coronary care unit, TRAS: tracheal aspirate, CSF: cerebrospinal fluid.

### Identification and validation of *A. baumannii* isolates

All 128 isolates were identified and confirmed as *A. baumannii* with 99% probability based on the biochemical profiles of Vitek 2 system (BioMërieux, Marcy-LʹEtoile, France) and 16S rRNA sequencing. These isolates were sequenced and identified as *A. baumannii* with the presence of an amplification band at 892 bp. A known *A. baumannii* strain (MB10070709) was used as the positive control.

### Antimicrobial susceptibility testing

All 128 *A. baumannii* isolates were categorized as multidrug-resistant attributed to their resistance to three or more classes of antibiotics. All isolates (*n* = 128, 100%) were resistant to ampicillin and imipenem. The resistance rate to ceftazidime was 97.7% followed by ciprofloxacin (97%), gentamicin (93%), and amikacin (92.2%). However, a small number of isolates that were resistant to imipenem remained susceptible to amikacin (7.8%), gentamicin (7.0%), ciprofloxacin (3.0%), and ceftazidime (2.3%). The main source of imipenem-resistant *A. baumannii* was tracheal aspirate (37.5%), and these *A. baumannii* were predominantly isolated from HDW (39.84%). Data on the susceptibility (zone diameter, mm/MIC, μg/mL) of all *A. baumannii* isolates against antibiotic used were included in the supplemental material (Table S2).

### Detection of antibiotic resistance genes by PCR amplification

#### Class D β-lactamases (*bla*_OXA-23_ and *bla*_OXA-24_)

The *bla*_OXA-23_ gene was represented by an amplification band at 606 bp. The gene was present in 99.22% of *A. baumannii* isolates and resides in both chromosomal and plasmid DNA. However, the *bla*_OXA-24_ gene was absent in both chromosomal and plasmid DNA among the *A. baumannii* isolates in the present study.

#### Class B β-lactamases (*bla*_VIM_ and *bla*_IMP_)

**Fig 1:**
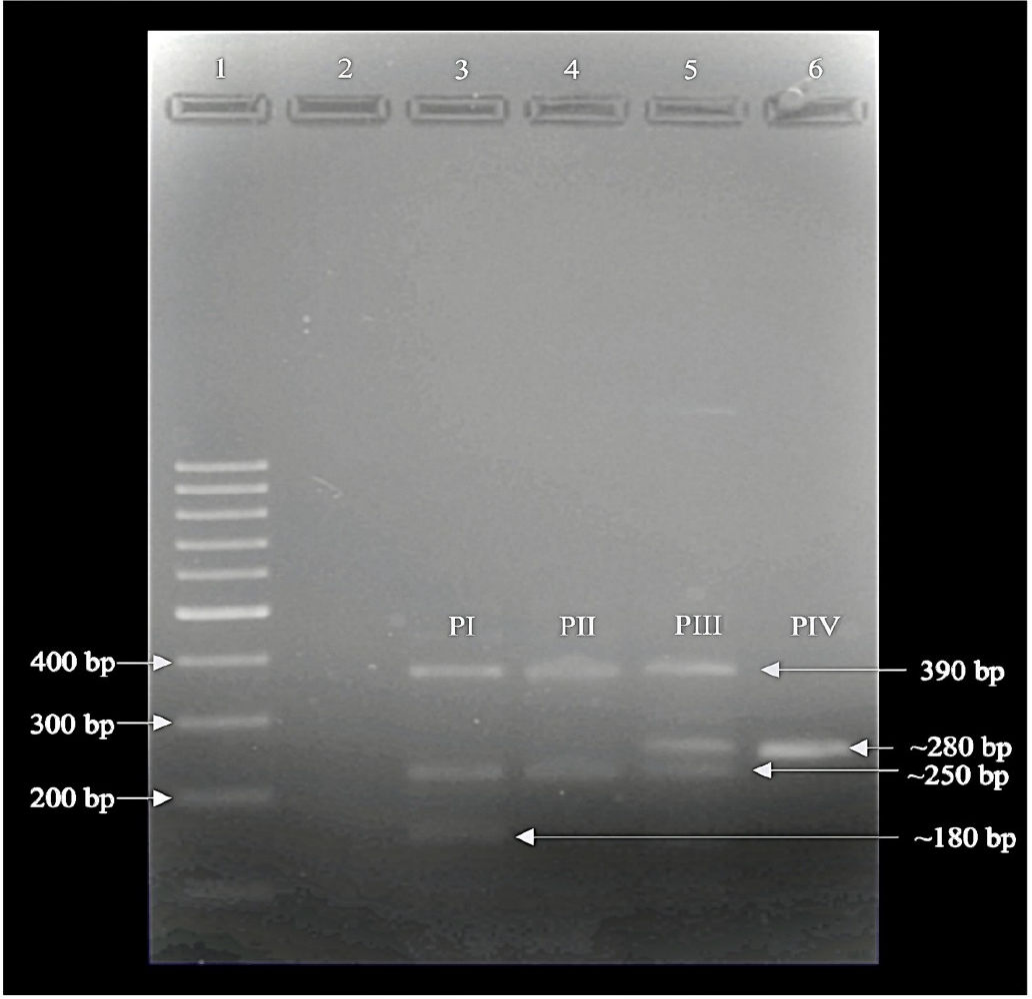
PCR amplification of *bla*_VIM_ gene in plasmid DNA of representative isolates of *A. baumannii*. Lane 1: 100 bp DNA ladder (Thermoscientific, USA); lane 2: negative control; lane 3: isolate 25 (PI, pattern I); lane 4: isolate 77 (PII, pattern II); lane 5: isolate 88 (PIII, pattern III); lane 6: isolate 101 (PIV, pattern IV).

The *bla*_VIM_ gene was detected in 99%–100% of *A. baumannii* isolates in both chromosomal and plasmid DNA at 390 bp. However, PCR amplification of the *bla*_VIM_ gene in the plasmid DNA formed four different band patterns among the *A. baumannii* isolates (Fig. 1). None of the *A. baumannii* isolates harbored *bla*_IMP_ gene either in chromosomal DNA or plasmid DNA.

Figure 1 illustrates the multiple band patterns (PI, PII, PIII, and PIV) of PCR amplification of the *bla*_VIM_ gene in the representative *A. baumannii* isolates, which correlates with their distinctive antibiotic susceptibility profile in Table 2.

**TABLE 2:** The antibiotic susceptibility profile of the representative *A. baumannii* isolates^*a*^

Isolate no.	Zone diameter (mm)/susceptibility/MIC (μg/mL)						PCR
	CAZ	AN	GM	AM	CIP	IP	Band pattern type
25	6, R, >128	6, R, >256	10, R, 24	6, R	6, R, >20	6, R, >24	P1
77	20, S, 10	21, S, 4	18, S, 4	6, R	27, S, 0.25	9, R, >24	PII
88	6, R, >128	6, R, >256	6, R, >48	6, R	6, R, >20	10, R, 20	PIII
101	6, R, >128	24, S, 2	15, S, 6	6, R	25, S, 0.5	13, R, 10	PIV

^*a*^ CAZ, ceftazidime; AN, amikacin; GM, gentamicin; AM, ampicillin; CIP, ciprofloxacin; and IP, imipenem.

#### Class C β-lactamases (*bla*_ADC_)

In the present study, the prevalence rate of *bla*_ADC_ gene is 98.44% in chromosomal DNA and 99.22% in the plasmid. Although isolate 101 exhibited resistance to ceftazidime, it did not harbor the *bla*_ADC_ gene. On the other hand, isolates 17, 43, and 77 harbored the *bla*_ADC_ gene despite being sensitive to ceftazidime.

### Insertion sequence IS*Aba1* upstream of *bla*_OXA-23_ gene

The insertion sequence IS*Aba1*/*bla*_OXA-23_ gene is responsible for the overproduction of the *bla*_OXA-23_ gene in *A. baumannii*. It was observed that 99.22% of the *A. baumannii* isolates produced IS*Aba1* upstream of the *bla*_OXA-23_ gene.

### PCR sequencing analysis

DNA sequencing analysis of the antibiotic resistance genes among *A. baumannii* isolates revealed two types of mutation involved, including point and frameshift mutations. Table 3 outlines the mutations that occurred in several nucleotides of antibiotic resistance genes with the type of mutation when compared to the reference gene sequence in the GenBank.

**TABLE 3:** Mutation occurred in the antibiotic resistance genes in comparison to the reference gene sequence from the database^*a*^

Antibiotic resistance gene	Percentage of DNA homology	Number of nucleotide changes with the type of mutation	
		Chromosome	Plasmid
*bla*_OXA-23_	98–99	3 PM, 8 FM	3 PM, 3 FM
IS*Aba*1/*bla*_OXA-23_	89	26 PM, 37 FM	–^*b*^
*bla*_ADC_	99	3 PM, 9 FM	3 PM, 5FM
*bla*_VIM_	99	3 PM	3 PM

^*a*^ PM, point mutation; FM, frameshift mutation.

^*b*^ –, No mutation detected.

### Multi-locus sequence typing and phylogenetic analysis

**Fig 2:**
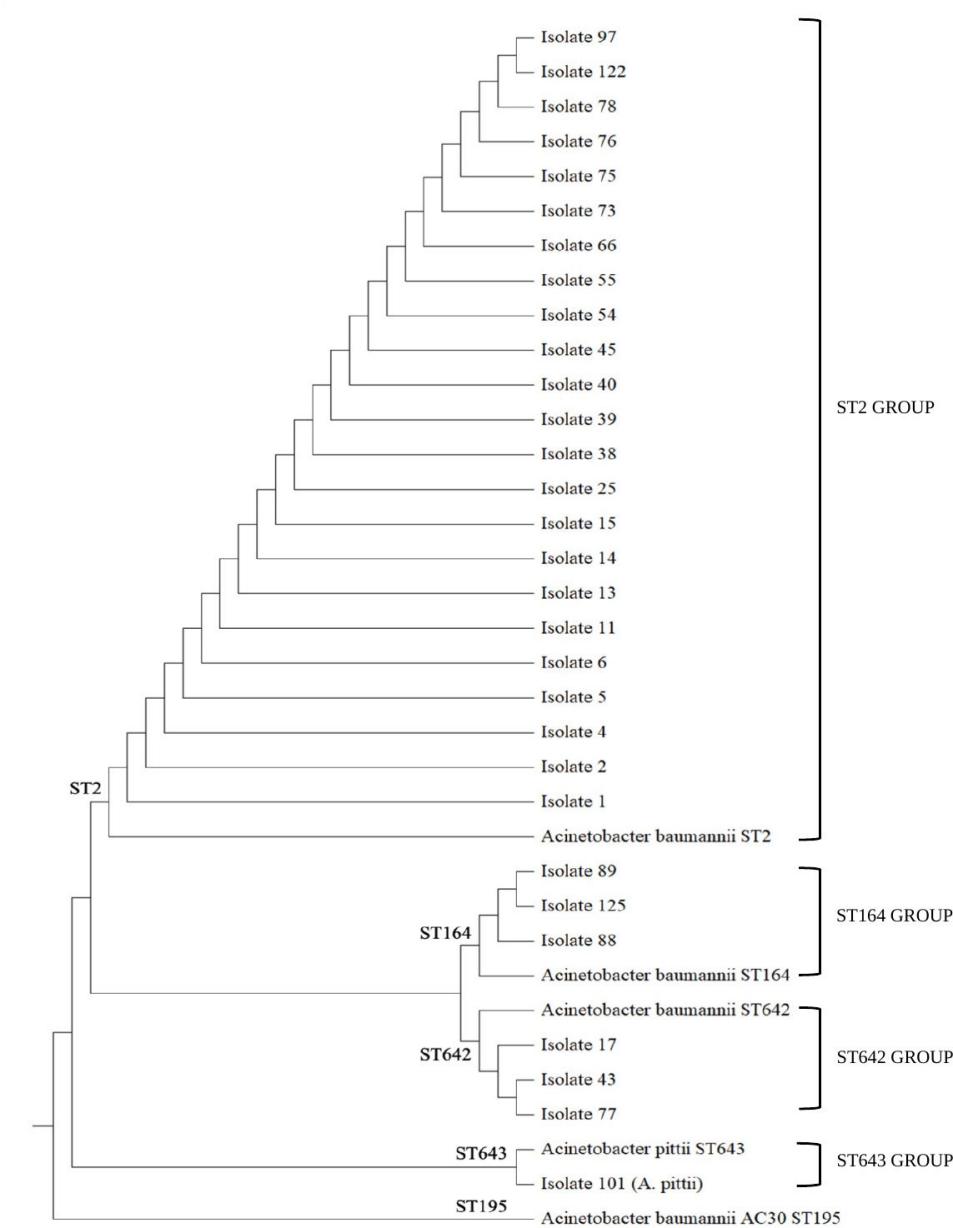
Phylogenetic tree of 30 MDR *A. baumannii* isolates with reference sequences of different *A. baumannii* STs from GenBank.

Seven housekeeping genes were used to confirm the identity of *A. baumannii* sequence type based on their allele numbers as attached in the supplementary section (S3). The genomic sequences of each *A. baumannii* ST were compared to the established STs, locally (ST195) and internationally (ST2, ST164, ST642, and ST643) to study the genetic relationship among all isolates. The phylogenetic tree (Fig. 2) shows the total number of 30 *A. baumannii* isolates clustered based on their genetic sequence that corresponds to specific ST. The most prevalent ST in this study was ST2 (76.67%). ST642 (10%), ST164 (10%), and ST643 (3.33%) were distinguished from ST2 by their genetic differences, which indicate different evolutionary lineages. The ST195 was included in this tree as a reference strain. The distribution and antibiotic resistance information of *A. baumannii* isolates for each ST are summarized in Table 4.

**TABLE 4:** Summary of distribution and antibiotic resistance details of *A. baumannii* isolates in different STs^*a*^

ST	Location	Specimen type	Detected antibiotic resistance gene	Antibiotic susceptibility pattern and PCR band pattern
ST2, 23 (76.67%)	HDW, 10 (43.48%), ICU, 7 (30.43%), CCU, 1 (4.35%), NICU, 2 (8.70%), general wards, 3 (13.04%)	TRAS, 9 (39.14%), BAL, 4 (17.38%), Blood, 4 (17.38%), CSF, 2 (8.70%), Urine, 2 (8.70%), Sputum, 1 (4.35%), Pus, 1 (4.35%)	*bla*_OXA-23_, *bla*_VIM_, *bla*_ADC,_ IS*Aba1/bla*_OXA-23_	Resistant to all antibiotics tested (PI)
ST164, 3 (10%)	General wards, 2 (66.67%), HDW, 1 (33.33%)	Tissue, 2 (66.67%), Aqueous fluid, 1 (33.33%)	*bla*_OXA-23_, *bla*_VIM_, *bla*_ADC,_ IS*Aba1/bla*_OXA-23_	Resistant to all antibiotics tested (PIII)
ST642, 3 (10%)	ICU, 1 (33.33%), HDW, 1 (33.33%), General wards, 1 (33.33%)	TRAS, 1 (33.33%), Blood, 2 (66.67%)	*bla*_OXA-23_, *bla*_VIM_, *bla*_ADC,_ IS*Aba1/bla*_OXA-23_	Sensitive to all antibiotics tested except ampicillin and imipenem (PII)
ST643, 1 (3.33%) (*Acinetobacter pittii*)	General wards, 1 (33.33%)	Blood, 1 (100%)	None except *bla*_VIM_	Sensitive to all antibiotics tested except ceftazidime, ampicillin, and imipenem (PIV)
ST195	Tertiary hospital in Terengganu	NA	*bla*_OXA-23_ and *bla*_ADC_	Resistant to all antibiotics tested similarly in this study (26).

^*a*^ NA, data not available.

## DISCUSSION

The emergence of various antibiotic resistance in *A. baumannii* has become a significant threat to the healthcare system. To date, most clinical *A. baumannii* isolates were classified and reported as MDR (13). Infections caused by MDR *A. baumannii* were frequently related to systems or organs that consist of high fluid content, including respiratory and urinary tract systems, cerebrospinal fluid, and peritoneal fluid (27). Findings in the present study were in concordance with the above-mentioned report as the majority of MDR *A. baumannii* was isolated from TRAS (37.5%) and BAL (13.28%). The ability of *A. baumannii* to adhere and colonize the surfaces in the hospital environment increases the risk of infection among debilitated patients who need to undergo certain medical procedures or interventions (28).

*A. baumannii* acquires resistance genes through plasmid, which can be transferred from one bacterium to another resulting in resistance to multiple antibiotics. Inactivating enzymes such as β-lactamases are responsible for bacterial resistance toward various antibiotics, which include carbapenem, penicillin, and cephalosporin (29). In this study, most of the isolates exhibited resistance to more than three classes of antibiotics, thus these isolates were considered MDR *A. baumannii*. Furthermore, all isolates (100%) were resistant to carbapenem and ampicillin, with more than 90% of isolates being resistant to ceftazidime (97.7%), amikacin (92.2%), gentamicin (93.0%), and ciprofloxacin (97.0%), respectively.

Aminoglycoside resistance, including gentamicin (93.0%) and amikacin (92.2%) in *A. baumannii* isolates, was higher than the reported national prevalence rate (6); nonetheless, the pattern of increasing resistance rate corresponded not only to the national report but also to other previous studies conducted in Malaysia (30, 31). Reports from other countries, such as China, Turkey, and Poland, showed an increasing trend of gentamicin resistance, from 64.8% in 2020 to 79.2% in 2021, from 25% to 100% from 2016 to 2021, and from 42% in 2017 to 61% in 2022, respectively (32–34). For amikacin resistance, isolates from Turkey showed a similar rising pattern of resistance rate ranging from 12.5% to 100% between 2016 and 2021, while 58.6% and 71% were reported in Iran and Poland, respectively (33–35). The resistance rate for ciprofloxacin (97%) was found to be higher in this study and in agreement with reports from Iran and Turkey (13, 36). In addition to that, all *A. baumannii* isolates were resistant to imipenem, and this corresponds to the escalating imipenem resistance rate reported by NSAR (6) (62.4%) (6) and other studies as well (9, 31). These data align with the imipenem resistance rate of *A. baumannii* in Vietnam, which was 91.6% between 2012 and 2014 (37). Class D β-lactamase, encoded by *bla*_OXA-23_ gene, was reported as the major contributing factor to carbapenem resistance (10, 38). The upregulation of the *bla*_OXA-23_ gene is due to the presence of IS*Aba*1 upstream of the gene, which serves as a strong promoter sequence (39). Our result is in parallel to earlier reports, as 99.2% of *A. baumannii* isolates were *bla*_OXA-23_ producers, and the gene was detected in both chromosomal and plasmid DNA (13, 14, 40). Moreover, these isolates also had IS*Aba*1 located upstream of the gene. Other than the *bla*_OXA-23_ gene, *bla*_VIM_ and *bla*_IMP_ genes in Class B metallo-β-lactamases are related to carbapenem resistance in *A. baumannii* (41). Although the *bla*_NDM_ gene is commonly studied due to its global significance, it was excluded from this study because of its low prevalence in Malaysia (42). In the present study, there was a high occurrence of the *bla*_VIM_ gene in *A. baumannii* isolates detected in both chromosomal and plasmid DNA. The amplification of the *bla*_VIM_ gene in plasmid DNA resulted in four distinct PCR band patterns, each corresponding to unique antibiotic susceptibility profiles, suggesting varying mechanisms of antibiotic resistance involved. The DNA analysis of the *bla*_VIM_ gene at 390 bp showed 99% homology with the two-component system of the histidine kinase (BaeS) gene. The BaeSR system plays an important role in regulating the efflux pump in *A. baumannii* (43). The overproduction of efflux pumps in *A. baumannii* is directly linked to the development of carbapenem resistance (16). In this study, isolates 17, 43, and 77 that belong to pattern type II (PII) were resistant to ampicillin and imipenem but remained susceptible to other antibiotics tested. Interestingly, this was not found in two previous studies of *bla*_VIM_ gene detection in Malaysia (9, 30). Previously, there was no evidence of using the *bla*_VIM_ gene as a basis for bacterial typing, but in this study, the PCR band patterns were used as one of the selection criteria for the next analysis. *Acinetobacter* spp. is known to encode *Acinetobacter*-derived AmpC cephalosporinases in the chromosomal DNA, while the plasmid-mediated AmpC beta-lactamases have also been reported persistently (44). In the present study, 98%–99% of *A. baumannii* isolates carried the *bla*_ADC_ gene in both chromosomal and plasmid DNA, which corresponds to the findings by researchers at UKMMC, Kuala Lumpur, Malaysia, where 93.7% of their isolates harbored the *bla*_ADC_ gene (9).

When compared to the published sequences of *A. baumannii* in the GenBank, the studied genes (*bla*_OXA-23_, IS*Aba*1/*bla*_OXA-23_, *bla*_ADC,_ and *bla*_VIM_) exhibited various mutations, including the addition and deletion of DNA nucleotide, frameshift and point mutations within the chromosome, and plasmid DNA. The alteration of genomic sequences was crucial to sustain the evolution of acquired resistance genes to adapt and survive against selective pressure (45).

In this study, the ST2 was the main cluster of MDR *A. baumannii* isolates existing within the hospital, and this is consistent with the epidemiological reports worldwide (46–51). The finding of plasmid-mediated resistance genes that encode for *bla*_OXA-23_, *bla*_ADC^,^_ and *bla*_VIM_ in *A. baumannii* results in rapid dissemination of antibiotic resistance genes among the strains or other bacteria in the hospital environments (10, 52, 53). The ST2 clone was associated with high resistance to carbapenem conferred by *bla*_OXA-23_ and IS*Aba*1 upstream of the *bla*_OXA-23_ gene. The ability of *A. baumannii* to survive under selective pressure attributed to the high usage of carbapenem leads to *A. baumannii* outbreak with the possibility of clonal expansion (48). The occurrence of ST164 of *A. baumannii* is associated with the presence of the *bla*_OXA-23_ gene, hence producing resistance to carbapenem, gentamicin, cephalosporin, and ciprofloxacin (49, 54). This corresponds to our finding as ST164 *A. baumannii* isolates in the present study were resistant to the respective antibiotics and primarily associated with the *bla*_OXA-23_ gene. Furthermore, this ST164 *A. baumannii* produced *bla*_VIM_, *bla*_ADC,_ and IS*Aba*1 upstream of *bla*_OXA-23_ gene that is responsible for carbapenem and cephalosporin resistance. In this study, *A. baumannii* isolates that belong to this clone were isolated from the general wards (isolates 88 and 89) and HDW (isolate 125). These findings strongly indicate that the same strain of *A. baumannii* from the general wards has been transmitted to HDW or circulating between these two locations. The transmission may occur via contaminated hands of healthcare workers (HCWs), inanimate objects, or direct contact between HCWs and patients. According to the multiple sequence analysis, *A. baumannii* isolates found in the HDW were genetically similar to the *A. baumannii* isolates in ICU, CCU, and other wards. The ST642 *A. baumannii* was reported to confer a high resistance rate to imipenem, ceftazidime, amikacin, and ciprofloxacin via the *bla*_OXA-23_ gene (55). In Indonesia and Pakistan, the ST642 *A. baumannii* carried *bla*_OXA-23_ and *aphA*6 and *aad*B genes, which were responsible for carbapenem and aminoglycoside resistance, respectively (56, 57). On the contrary, in this study, it was observed that three isolates (isolates 17, 43, and 77) exhibited susceptibility to ceftazidime, amikacin, gentamicin, and ciprofloxacin but were resistant to imipenem and ampicillin. Despite their susceptibility to most antibiotics, these isolates produce oxacillinase, cephalosporinase, and metallo-β-lactamase enzymes owing to the detection of *bla*_OXA-23_, *bla*_ADC_, *bla*_VIM^,^_ and IS*Aba*1 upstream of the *bla*_OXA-23_ gene. The variation in antibiotic susceptibility profiles among the ST642 *A. baumannii* suggests that the isolates have been exposed to selective pressure within the hospital environment. Additionally, the diversity in resistance patterns might indicate the association of other resistance mechanisms such as overexpression of efflux pumps (AdeABC) or porin modifications (loss of CarO gene) (16, 17, 58) in *A. baumannii*. Further investigation at the genetic and proteomic levels could clarify the contribution of these factors to the overall resistance profile. The ST642 *A. baumannii* isolates were prevalent in three different locations, namely ICU, HDW, and general wards. This observation reflects the possibility of cross-contamination from HCW to patients within these respective locations.

Isolate 101, which belongs to ST643, was isolated from blood in a general ward and was initially identified as *A. baumannii* by Vitek 2 system. Upon comparing its *rpoB* gene with known DNA sequences in the GenBank, isolate 101 exhibited 99% similarity to the *Acinetobacter pittii* strain with accession number CP043052.1 that was isolated from a urine sample of a patient in Hangzhou, China. This occurrence of misidentification of *A. pittii* is supported by Ang et al. (59), where their ST119 *A. pittii* isolate was initially identified as *A. baumannii* using the API 20NE system but was later confirmed as *A. pittii* through *rpoB* gene sequencing. The 16S rRNA sequencing was reported to be insufficient for distinguishing *Acinetobacter* at the species level due to its low polymorphic nature in the variable region (60).

The present study involves testing isolates obtained from both sterile and non-sterile specimens from one of the tertiary care centers in Malaysia; hence, this might not be an actual representative of Malaysian isolates. Furthermore, the phylogenetic analysis performed was limited to only 30 MDR *A. baumannii* isolates. The selection of isolates was based on their distinct antibiotic resistance profiles and the presence of antibiotic resistance genes, which may reveal the underlying genetic differences among the *A. baumannii*. If all 128 isolates were investigated by MLST, more ST discoveries might be achieved for national and sentinel surveillance, and the precise location of the antibiotic resistance cassette can be determined using whole-genome sequencing.

Our findings were consistent with the global surveillance data, whereby *bla*_OXA-23_ was the most common carbapenemase-encoding gene among the MDR *A. baumannii* isolates (13, 14). There were three sequence types (ST2, ST164, and ST642) identified, with both ST164 and ST642 being unique to Malaysian isolates. Thus far, sequence types in Malaysia include ST92, ST195, ST208, ST938, ST1418, ST1947, ST1948, and ST426 for Oxford scheme (26, 42), while ST374, ST46, ST220, ST360, and ST739 for Pasteur scheme (61). The ST2 was the most prevalent clone circulating in ICU, CCU, HDW, NICU, and general wards, suggesting the transmission of *A. baumannii* among the healthcare workers, fomites, and -patients has occurred. The association between ST2 with the carbapenem resistance and *bla*_OXA-23_, IS*Aba1* upstream of the *bla*_OXA-23_ gene, was evident. Interestingly, our isolates denoted ST642 demonstrated a contrast of AST’s profile, suggesting that genetic evolution occurred, which enabled them to strive for selective pressure. Our study revealed that isolates with different resistance profiles correspond to different STs, suggesting that genetic mutation among these isolates altered according to their resistance patterns. This approach may provide insight into the important implications for epidemiological tracking and treatment strategies.

In addition, the findings in this study align with national and global trends of *A. baumannii*. The data emphasize the importance of sentinel surveillance to trace the emergence of novel resistance mechanisms or the development of new *A. baumannii* strains worldwide. It is worth conducting a longitudinal study design with a similar clinical setting over a defined period to capture potential changes in the antibiotic resistance profile of the *A. baumannii* isolates. Incorporating whole-genome sequencing on the sequential isolates will allow a comprehensive analysis of the presence and evolution of resistance genes over time. These data provide insight into the genetic diversity, resistance mechanisms, and transmission patterns of *A. baumannii*, contributing to more effective outbreak management and prevention in the future.

## SUPPLEMENTAL MATERIAL

Supplemental material spectrum.00872-24-s0001.docx Sequence types (STs) identification of *A. baumannii* isolates, (n=30).

Table S1 spectrum.00872-24-s0002.docx List of primers sequence for antibiotic resistance gene.

Table S2 spectrum.00872-24-s0003.docx Resistance rate of *A. baumannii* isolates by disk-diffusion and E-test using CLSI interpretive criteria.

ASM does not own the copyrights to Supplemental Material that may be linked to, or accessed through, an article. The authors have granted ASM a non-exclusive, world-wide license to publish the Supplemental Material files. Please contact the corresponding author directly for reuse.
