# Supplementary material for: Genetic profiling of multidrug-resistant Acinetobacter baumannii from a tertiary care center in Malaysia
Source: Microbiol Spectr. 2024 Dec 20;13(2):e00872-24. doi: 10.1128/spectrum.00872-24 (PMC11792510; doi:10.1128/spectrum.00872-24)
Supplement: Supplemental material — Sequence types (STs) identification of A. baumannii isolates, (n=30). [file spectrum.00872-24-s0001.docx]

**S3: Sequence types (STs) Identification of *A. baumannii* Isolates, (n=30)**

| Isolate No. | ST | Allele No. | | | | | | |
| --- | --- | --- | --- | --- | --- | --- | --- | --- |
|  |  | ***cpn60*** | ***fusA*** | ***gltA*** | ***pyrG*** | ***recA*** | ***rplB*** | ***rpoB*** |
| 1 | 2 | 2 | 2 | 2 | 2 | 2 | 2 | 2 |
| 2 | 2 | 2 | 2 | 2 | 2 | 2 | 2 | 2 |
| 4 | 2 | 2 | 2 | 2 | 2 | 2 | 2 | 2 |
| 5 | 2 | 2 | 2 | 2 | 2 | 2 | 2 | 2 |
| 6 | 2 | 2 | 2 | 2 | 2 | 2 | 2 | 2 |
| 11 | 2 | 2 | 2 | 2 | 2 | 2 | 2 | 2 |
| 13 | 2 | 2 | 2 | 2 | 2 | 2 | 2 | 2 |
| 14 | 2 | 2 | 2 | 2 | 2 | 2 | 2 | 2 |
| 15 | 2 | 2 | 2 | 2 | 2 | 2 | 2 | 2 |
| 17 | 642 | 1 | 1 | 1 | 1 | 9 | 1 | 1 |
| 25 | 2 | 2 | 2 | 2 | 2 | 2 | 2 | 2 |
| 38 | 2 | 2 | 2 | 2 | 2 | 2 | 2 | 2 |
| 39 | 2 | 2 | 2 | 2 | 2 | 2 | 2 | 2 |
| 40 | 2 | 2 | 2 | 2 | 2 | 2 | 2 | 2 |
| 43 | 642 | 1 | 1 | 1 | 1 | 9 | 1 | 1 |
| 45 | 2 | 2 | 2 | 2 | 2 | 2 | 2 | 2 |
| 54 | 2 | 2 | 2 | 2 | 2 | 2 | 2 | 2 |
| 55 | 2 | 2 | 2 | 2 | 2 | 2 | 2 | 2 |
| 66 | 2 | 2 | 2 | 2 | 2 | 2 | 2 | 2 |
| 73 | 2 | 2 | 2 | 2 | 2 | 2 | 2 | 2 |
| 75 | 2 | 2 | 2 | 2 | 2 | 2 | 2 | 2 |
| 76 | 2 | 2 | 2 | 2 | 2 | 2 | 2 | 2 |
| 77 | 642 | 1 | 1 | 1 | 1 | 9 | 1 | 1 |
| 78 | 2 | 2 | 2 | 2 | 2 | 2 | 2 | 2 |
| 88 | 164 | 40 | 3 | 7 | 2 | 40 | 4 | 4 |
| 89 | 164 | 40 | 3 | 7 | 2 | 40 | 4 | 4 |
| 97 | 2 | 2 | 2 | 2 | 2 | 2 | 2 | 2 |
| 101 | 643 | 45 | 58 | 44 | 10 | 218 | 18 | 20 |
| 122 | 2 | 2 | 2 | 2 | 2 | 2 | 2 | 2 |
| 125 | 164 | 40 | 3 | 7 | 2 | 40 | 4 | 4 |
