# Supplementary material for: Genetic profiling of multidrug-resistant Acinetobacter baumannii from a tertiary care center in Malaysia
Source: Microbiol Spectr. 2024 Dec 20;13(2):e00872-24. doi: 10.1128/spectrum.00872-24 (PMC11792510; doi:10.1128/spectrum.00872-24)
Supplement: Table S1 — List of primers sequence for antibiotic resistance gene. [file spectrum.00872-24-s0002.docx]

**S1: Primers Sequence for Antibiotic Resistance Gene**

| **Primer name** | **Primer sequence**  **(5’ to 3’)** | **Annealing Temperature/Ta (°C)** | **Reference** |
| --- | --- | --- | --- |
| *bla*_OXA-23_ | F- TCTGGTTGTACGGTTCAGC  R- AGTCTTTCCAAAAATTTTG | 50.0 | (9, 62) |
| *bla*_OXA-24_ | F- GGTTAGTTGGCCCCCTTAAA  R- AGTTGAGCGAAAAGGGGATT | 63.5 | (25) |
| *bla*_IMP_ | F- GTTTATGTTCATACWTCG  R- GGTTTAAYAAAACAACCA | 51.5 | (9, 62) |
| *(bla*_VIM_ | F- GATGGTGTTTGGTCGCATA  R- CGAATGCGCAGCACCAG | 50.0 | (25) |
| *bla*_ADC_ | F- ATGCGATTTAAAAAAATTTCTTGT  R- TTATTTCTTTATTGCATTCAG | 56.0 | (9) |
| IS*Aba*1 of *bla*_OXA-23_ | F-GATCCCTCTGTACACGAYAAATTTC  R- AGTCTTTCCAAAAATTTTG | 63.2 | (9, 63) |
