# Supplementary material for: Genetic profiling of multidrug-resistant Acinetobacter baumannii from a tertiary care center in Malaysia
Source: Microbiol Spectr. 2024 Dec 20;13(2):e00872-24. doi: 10.1128/spectrum.00872-24 (PMC11792510; doi:10.1128/spectrum.00872-24)
Supplement: Table S2 — Resistance rate of A. baumannii isolates by disk-diffusion and E-test using CLSI interpretive criteria. [file spectrum.00872-24-s0003.docx]

**S2: Resistance rate of *A. baumannii* isolates by disk-diffusion and E-test using CLSI Interpretive Criteria**

| Isolate No | Zone Diameter (mm)/Susceptibility/MIC (μg/mL) | | | | | | E-Test (μg/mL) | | |
| --- | --- | --- | --- | --- | --- | --- | --- | --- | --- |
|  | **CAZ** | **AN** | **GM** | **AM** | **CIP** | **IPM** | | **IP** |  |
| 1 | 6, R, >128 | 6, R, >256 | 7, R, >48 | 6, R | 6, R, >20 | 6, R, >24 | | >24, R |  |
| 2 | 6, R, >128 | 23, S, 2 | 15, S, 6 | 6, R | 6, R, >20 | 6, R, >24 | | >24, R |  |
| 3 | 6, R, >128 | 6, R, >256 | 6, R, >48 | 6, R | 6, R, >20 | 6, R, >24 | | >24, R |  |
| 4 | 6, R, >128 | 19, S, 8 | 10, R, 24 | 6, R | 6, R, >20 | 6, R, >24 | | >24, R |  |
| 5 | 6, R, >128 | 8, R, >256 | 10, R, 24 | 6, R | 6, R, >20 | 8, R, >24 | | >24, R |  |
| 6 | 6, R, >128 | 18, S, 10 | 15, S, 6 | 6, R | 6, R, >20 | 6, R, >24 | | >24, R |  |
| 7 | 6, R, >128 | 8, R, >256 | 7, R, >48 | 7, R | 6, R, >20 | 6, R, >24 | | >24, R |  |
| 8 | 6, R, >128 | 6, R, >256 | 10, R, 24 | 6, R | 6, R, >20 | 6, R, >24 | | >24, R |  |
| 9 | 6, R, >128 | 6, R, >256 | 6, R, >48 | 6, R | 6, R, >20 | 6, R, >24 | | >24, R |  |
| 10 | 6, R, >128 | 6, R, >256 | 8, R, >48 | 6, R | 6, R, >20 | 6, R, >24 | | >24, R |  |
| 11 | 6, R, >128 | 6, R, >256 | 6, R, >48 | 6, R | 6, R, >20 | 6, R, >24 | | >24, R |  |
| 12 | 6, R, >128 | 6, R, >256 | 6, R, >48 | 6, R | 6, R, >20 | 6, R, >24 | | >24, R |  |
| 13 | 6, R, >128 | 6, R, >256 | 8, R, >48 | 6, R | 6, R, >20 | 7, R, >24 | | >24, R |  |
| 14 | 6, R, >128 | 6, R, >256 | 12, R, 16 | 6, R | 6, R, >20 | 6, R, >24 | | >24, R |  |
| 15 | 6, R, >128 | 6, R, >256 | 6, R, >48 | 6, R | 6, R, >20 | 8, R, >24 | | >24, R |  |
| 16 | 6, R, >128 | 6, R, >256 | 7, R, >48 | 6, R | 6, R, >20 | 6, R, >24 | | >24, R |  |
| 17 | 19, S, 12 | 23, S, 2 | 20, S, 2 | 6, R | 29, S, <0.125 | 6, R, >24 | | >24, R |  |
| 18 | 6, R, >128 | 6, R, >256 | 6, R, >48 | 6, R | 6, R, >20 | 8, R, >24 | | >24, R |  |
| 19 | 6, R, >128 | 6, R, >256 | 8, R, >48 | 6, R | 6, R, >20 | 6, R, >24 | | >24, R |  |
| 20 | 6, R, >128 | 13, R, 48 | 10, R, 24 | 6, R | 6, R, >20 | 6, R, >24 | | >24, R |  |
| 21 | 6, R, >128 | 14, R, 48 | 10, R, 24 | 7, R | 6, R, >20 | 6, R, >24 | | >24, R |  |
| 22 | 6, R, >128 | 6, R, >256 | 6, R, >48 | 8, R | 6, R, >20 | 6, R, >24 | | >24, R |  |
| 23 | 6, R, >128 | 12, R, 64 | 10, R, 24 | 6, R | 9, R, >20 | 6, R, >24 | | >24, R |  |
| 24 | 6, R, >128 | 14, R, 48 | 10, R, 24 | 6, R | 6, R, >20 | 6, R, >24 | | >24, R |  |
| 25 | 6, R, >128 | 6, R, >256 | 10, R, 24 | 6, R | 6, R, >20 | 6, R, >24 | | >24, R |  |
| 26 | 6, R, >128 | 6, R, >256 | 7, R, >48 | 6, R | 6, R, >20 | 7, R, >24 | | >24, R |  |
| 27 | 6, R, >128 | 6, R, >256 | 6, R, >48 | 6, R | 6, R, >20 | 6, R, >24 | | >24, R |  |
| 28 | 6, R, >128 | 6, R, >256 | 6, R, >48 | 6, R | 6, R, >20 | 6, R, >24 | | >24, R |  |
| 29 | 6, R, >128 | 14, R, 48 | 11, R, 20 | 6, R | 6, R, >20 | 6, R, >24 | | >24, R |  |
| 30 | 6, R, >128 | 7, R, >256 | 6, R, >48 | 6, R | 6, R, >20 | 8, R, >24 | | >24, R |  |
| 31 | 6, R, >128 | 6, R, >256 | 10, R, 24 | 6, R | 6, R, >20 | 6, R, >24 | | >24, R |  |
| 32 | 9, R, >128 | 6, R, >256 | 6, R, >48 | 6, R | 6, R, >20 | 7, R, >24 | | >24, R |  |
| 33 | 6, R, >128 | 6, R, >256 | 6, R, >48 | 6, R | 6, R, >20 | 6, R, >24 | | >24, R |  |
| 34 | 6, R, >128 | 6, R, >256 | 6, R, >48 | 6, R | 6, R, >20 | 7, R, >24 | | >24, R |  |
| 35 | 6, R, >128 | 6, R, >256 | 6, R, >48 | 6, R | 6, R, >20 | 6, R, >24 | | >24, R |  |
| 36 | 6, R, >128 | 6, R, >256 | 6, R, >48 | 9, R | 6, R, >20 | 6, R, >24 | | >24, R |  |
| 37 | 6, R, >128 | 6, R, >256 | 6, R, >48 | 6, R | 6, R, >20 | 6, R, >24 | | >24, R |  |
| 38 | 6, R, >128 | 21, S, 4 | 16, S, 6 | 6, R | 6, R, >20 | 6, R, >24 | | >24, R |  |
| 39 | 6, R, >128 | 26, S, <1 | 21, S, 2 | 6, R | 6, R, >20 | 6, R, >24 | | >24, R |  |
| 40 | 6, R, >128 | 24, S, 2 | 18, S, 4 | 6, R | 6, R, >20 | 6, R, >24 | | >24, R |  |
| 41 | 6, R, >128 | 6, R, >256 | 6, R, >48 | 6, R | 6, R, >20 | 6, R, >24 | | >24, R |  |
| 42 | 6, R, >128 | 6, R, >256 | 6, R, >48 | 6, R | 6, R, >20 | 6, R, >24 | | >24, R |  |
| 43 | 19, S, 12 | 22, S. 4 | 18, S, 4 | 6, R | 28, S, 0.25 | 9, R, >24 | | >24, R |  |
| 44 | 8, R, >128 | 6, R, >256 | 12, R, 16 | 6, R | 6, R, >20 | 6, R, >24 | | >24, R |  |
| 45 | 6, R, >128 | 7, R, >256 | 6, R, >48 | 6, R | 6, R, >20 | 6, R, >24 | | >24, R |  |
| 46 | 6, R, >128 | 7, R, >256 | 6, R, >48 | 6, R | 6, R, >20 | 6, R, >24 | | >24, R |  |
| 47 | 6, R, >128 | 6, R, >256 | 6, R, >48 | 6, R | 6, R, >20 | 9, R, >24 | | >24, R |  |
| 48 | 6, R, >128 | 10, R, 128 | 11, R, 20 | 6, R | 6, R, >20 | 8, R, >24 | | >24, R |  |
| 49 | 6, R, >128 | 8, R, >256 | 12, R, 16 | 6, R | 6, R, >20 | 6, R, >24 | | >24, R |  |
| 50 | 6, R, >128 | 10, R, 128 | 12, R, 16 | 6, R | 6, R, >20 | 6, R, >24 | | >24, R |  |
| 51 | 6, R, >128 | 14, R, 48 | 11, R, 20 | 6, R | 6, R, >20 | 6, R, >24 | | >24, R |  |
| 52 | 6, R, >128 | 6, R, >256 | 12, R, 16 | 6, R | 6, R, >20 | 6, R, >24 | | >24, R |  |
| 53 | 6, R, >128 | 6, R, >256 | 12, R, 16 | 6, R | 6, R, >20 | 6, R, >24 | | >24, R |  |
| 54 | 6, R, >128 | 6, R, >256 | 12, R, 16 | 6, R | 6, R, >20 | 7, R, >24 | | >24, R |  |
| 55 | 6, R, >128 | 6, R, >256 | 12, R, 16 | 6, R | 6, R, >20 | 6, R, >24 | | >24, R |  |
| 56 | 6, R, >128 | 6, R, >256 | 6, R, >48 | 6, R | 6, R, >20 | 7, R, >24 | | >24, R |  |
| 57 | 6, R, >128 | 6, R, >256 | 6, R, >48 | 6, R | 6, R, >20 | 8, R, >24 | | >24, R |  |
| 58 | 6, R, >128 | 6, R, >256 | 6, R, >48 | 6, R | 6, R, >20 | 6, R, >24 | | >24, R |  |
| 59 | 6, R, >128 | 6, R, >256 | 6, R, >48 | 6, R | 6, R, >20 | 6, R, >24 | | >24, R |  |
| 60 | 6, R, >128 | 6, R, >256 | 6, R, >48 | 6, R | 6, R, >20 | 9, R, >24 | | >24, R |  |
| 61 | 6, R, >128 | 6, R, >256 | 6, R, >48 | 6, R | 6, R, >20 | 6, R, >24 | | >24, R |  |
| 62 | 6, R , >128 | 6, R, >256 | 6, R, >48 | 6, R | 6, R, >20 | 6, R, >24 | | >24, R |  |
| 63 | 6, R, >128 | 6, R, >256 | 12, R, 16 | 6, R | 6, R, >20 | 8, R, >24 | | >24, R |  |
| 64 | 6, R, >128 | 8, R, >256 | 12, R, 16 | 6, R | 6, R, >20 | 6, R, >24 | | >24, R |  |
| 65 | 6, R, >128 | 6, R, >256 | 12, R, 16 | 6, R | 6, R, >20 | 7, R, >24 | | >24, R |  |
| 66 | 6, R, >128 | 6, R, >256 | 6, R, >48 | 6, R | 6, R, >20 | 6, R, >24 | | >24, R |  |
| 67 | 6, R, >128 | 6, R, >256 | 8, R, >48 | 6, R | 6, R, >20 | 6, R, >24 | | >24, R |  |
| 68 | 6, R, >128 | 6, R, >256 | 12, R, 16 | 7, R | 6, R, >20 | 7, R, >24 | | >24, R |  |
| 69 | 6, R, >128 | 6, R, >256 | 12, R, 16 | 6, R | 6, R, >20 | 7, R, >24 | | >24, R |  |
| 70 | 6, R, >128 | 6, R, >256 | 6, R, >48 | 6, R | 6, R, >20 | 6, R, >24 | | >24, R |  |
| 71 | 6, R, >128 | 6, R, >256 | 6, R, >48 | 6, R | 6, R, >20 | 6, R, >24 | | >24, R |  |
| 72 | 6, R, >128 | 6, R, >256 | 6, R, >48 | 6, R | 6, R, >20 | 6, R, >24 | | >24, R |  |
| 73 | 6, R, >128 | 6, R, >256 | 6, R, >48 | 6, R | 6, R, >20 | 6, R, >24 | | >24, R |  |
| 74 | 6, R, >128 | 6, R, >256 | 6, R, >48 | 7, R | 6, R, >20 | 6, R, >24 | | >24, R |  |
| 75 | 6, R, >128 | 6, R, >256 | 6, R, >48 | 6, R | 6, R, >20 | 6, R, >24 | | >24, R |  |
| 76 | 6, R, >128 | 6, R, >256 | 6, R, >48 | 6, R | 6, R, >20 | 6, R, >24 | | >24, R |  |
| 77 | 20, S, 10 | 21, S, 4 | 18, S, 4 | 6, R | 27, S, 0.25 | 9, R, >24 | | >24, R |  |
| 78 | 6, R, >128 | 6, R, >256 | 6, R, >48 | 6, R | 6, R, >20 | 7, R, >24 | | >24, R |  |
| 79 | 6, R, >128 | 6, R, >256 | 6, R, >48 | 6, R | 6, R, >20 | 8, R, >24 | | >24, R |  |
| 80 | 6, R, >128 | 6, R, >256 | 6, R, >48 | 6, R | 6, R, >20 | 6, R, >24 | | >24, R |  |
| 81 | 6, R, >128 | 6, R, >256 | 6, R, >48 | 6, R | 6, R, >20 | 8, R, >24 | | >24, R |  |
| 82 | 6, R, >128 | 6, R, >256 | 6, R, >48 | 6, R | 6, R, >20 | 6, R, >24 | | >24, R |  |
| 83 | 6, R, >128 | 6, R, >256 | 6, R, >48 | 6, R | 6, R, >20 | 6, R, >24 | | >24, R |  |
| 84 | 6, R, >128 | 6, R, >256 | 6, R, >48 | 6, R | 6, R, >20 | 6, R, >24 | | >24, R |  |
| 85 | 6, R, >128 | 6, R, >256 | 6, R, >48 | 6, R | 6, R, >20 | 6, R, >24 | | >24, R |  |
| 86 | 6, R, >128 | 6, R, >256 | 6, R, >48 | 6, R | 6, R, >20 | 6, R, >24 | | >24, R |  |
| 87 | 6, R, >128 | 6, R, >256 | 6, R, >48 | 6, R | 6, R, >20 | 6, R, >24 | | >24, R |  |
| 88 | 6, R, >128 | 6, R, >256 | 6, R, >48 | 6, R | 6, R, >20 | 10, R, 20 | | >24, R |  |
| 89 | 6, R, >128 | 6, R, >256 | 6, R, >48 | 6, R | 6, R, >20 | 10, R, 20 | | >24, R |  |
| 90 | 6, R, >128 | 6, R, >256 | 6, R, >48 | 6, R | 6, R, >20 | 6, R, >24 | | >24, R |  |
| 91 | 6, R, >128 | 6, R, >256 | 6, R, >48 | 6, R | 6, R, >20 | 6, R, >24 | | >24, R |  |
| 92 | 6, R, >128 | 6, R, >256 | 12, R, 16 | 6, R | 6, R, >20 | 6, R, >24 | | >24, R |  |
| 93 | 6, R, >128 | 6, R, >256 | 6, R, >48 | 6, R | 6, R, >20 | 6, R, >24 | | >24, R |  |
| 94 | 6, R, >128 | 6, R, >256 | 6, R, >48 | 6, R | 6, R, >20 | 6, R, >24 | | >24, R |  |
| 95 | 6, R, >128 | 6, R, >256 | 6, R, >48 | 6, R | 8, R, >20 | 7, R, >24 | | >24, R |  |
| 96 | 6, R, >128 | 14, R, 48 | 12, R, 16 | 6, R | 7, R, >20 | 6, R, >24 | | >24, R |  |
| 97 | 6, R, >128 | 6, R, >256 | 6, R, >48 | 7, R | 6, R, >20 | 6, R, >24 | | >24, R |  |
| 98 | 6, R, >128 | 6, R, >256 | 10, R, 24 | 6, R | 6, R, >20 | 8, R, >24 | | >24, R |  |
| 99 | 6, R, >128 | 6, R, >256 | 8, R, >48 | 6, R | 6, R, >20 | 7, R, >24 | | >24, R |  |
| 100 | 6, R, >128 | 6, R, >256 | 9, R, 32 | 6, R | 6, R, >20 | 9, R, >24 | | >24, R |  |
| 101 | 6, R, >128 | 24, S, 2 | 15, S, 6 | 6, R | 25, S, 0.5 | 13, R, 10 | | 10, R |  |
| 102 | 6, R, >128 | 6, R, >256 | 12, R, 16 | 6, R | 6, R, >20 | 6, R, >24 | | >24, R |  |
| 103 | 6, R, >128 | 8, R, >256 | 6, R, >48 | 6, R | 6, R, >20 | 8, R, >24 | | >24, R |  |
| 104 | 6, R, >128 | 6, R, >256 | 6, R, >48 | 6, R | 6, R, >20 | 9, R, >24 | | >24, R |  |
| 105 | 6, R, >128 | 6, R, >256 | 6, R, >48 | 6, R | 6, R, >20 | 6, R, >24 | | >24, R |  |
| 106 | 6, R, >128 | 6, R, >256 | 6, R, >48 | 6, R | 6, R, >20 | 6, R, >24 | | >24, R |  |
| 107 | 6, R, >128 | 6, R, >256 | 12, R, 16 | 6, R | 6, R, >20 | 8, R, >24 | | >24, R |  |
| 108 | 6, R, >128 | 6, R, >256 | 12, R, 16 | 6, R | 6, R, >20 | 6, R, >24 | | >24, R |  |
| 109 | 6, R, >128 | 6, R, >256 | 6, R, >48 | 6, R | 13, R, 12 | 6, R, >24 | | >24, R |  |
| 110 | 6, R, >128 | 6, R, >256 | 6, R, >48 | 6, R | 6, R, >20 | 12, R, 12 | | >24, R |  |
| 111 | 6, R, >128 | 6, R, >256 | 9, R, 32 | 6, R | 6, R, >20 | 6, R, >24 | | >24, R |  |
| 112 | 6, R, >128 | 6, R, >256 | 10, R, 24 | 6, R | 6, R, >20 | 6, R, >24 | | >24, R |  |
| 113 | 6, R, >128 | 6, R, >256 | 10, R, 24 | 6, R | 6, R, >20 | 6, R, >48 | | >48, R |  |
| 114 | 6, R, >128 | 6, R, >256 | 6, R, >48 | 6, R | 6, R, >20 | 6, R, >24 | | 12, R |  |
| 115 | 6, R, >128 | 6, R, >256 | 6, R, >48 | 6, R | 6, R, >20 | 9, R, >24 | | >24, R |  |
| 116 | 6, R, >128 | 6, R, >256 | 6, R, >48 | 6, R | 6, R, >20 | 9, R, >24 | | >24, R |  |
| 117 | 6, R, >128 | 6, R, >256 | 6, R, >48 | 6, R | 6, R, >20 | 7, R, >24 | | 12, R |  |
| 118 | 6, R, >128 | 6, R, >256 | 6, R, >48 | 8, R | 6, R, >20 | 8, R, >24 | | >24, R |  |
| 119 | 6, R, >128 | 6, R, >256 | 6, R, >48 | 6, R | 6, R, >20 | 6, R, >24 | | >24, R |  |
| 120 | 6, R, >128 | 6, R, >256 | 6, R, >48 | 6, R | 6, R, >20 | 6, R, >24 | | >24, R |  |
| 121 | 6, R, >128 | 6, R, >256 | 6, R, >48 | 6, R | 6, R, >20 | 6, R, >24 | | >24, R |  |
| 122 | 6, R, >128 | 6, R, >256 | 6, R, >48 | 8, R | 6, R, >20 | 6, R, >24 | | 12, R |  |
| 123 | 6, R, >128 | 6, R, >256 | 6, R, >48 | 6, R | 6, R, >20 | 6, R, >24 | | >24, R |  |
| 124 | 6, R, >128 | 6, R, >256 | 6, R, >48 | 6, R | 6, R, >20 | 6, R, >24 | | >24, R |  |
| 125 | 6, R, >128 | 6, R, >256 | 6, R, >48 | 6, R | 6, R, >20 | 6, R, >24 | | >24, R |  |
| 126 | 6, R, >128 | 6, R, >256 | 6, R, >48 | 6, R | 6, R, >20 | 8, R, >24 | | >24, R |  |
| 127 | 6, R, >128 | 6, R, >256 | 6, R, >48 | 6, R | 6, R, >20 | 6, R, >24 | | 12, R |  |
| 128 | 6, R, >128 | 6, R, >256 | 7, R, >48 | 6, R | 6, R, >20 | 6, R, >24 | | 12, R |  |

Abbreviation: CAZ; ceftazidime, AN; amikacin, GM; gentamicin, AM; ampicillin, CIP; ciprofloxacin, IPM/IP; imipenem, R; resistant, and S; sensitive
